# Oxidation is an underappreciated post-translational modification in the regulation of immune responses associated with changes in phosphorylation

**DOI:** 10.3389/fimmu.2023.1244431

**Published:** 2023-09-22

**Authors:** Isabel Karkossa, Sabine Fürst, Henning Großkopf, Martin von Bergen, Kristin Schubert

**Affiliations:** ^1^ Department of Molecular Systems Biology, Helmholtz-Centre for Environmental Research - UFZ, Leipzig, Germany; ^2^ Institute of Biochemistry, Leipzig University, Leipzig, Germany; ^3^ German Centre for Integrative Biodiversity Research (iDiv) Halle-Jena-Leipzig, Leipzig, Germany

**Keywords:** THP-1, LPS, proteome, phosphoproteome, redoxome

## Abstract

Although macrophages are known to be affected by their redox status, oxidation is not yet a well-recognized post-translational modification (PTM) in regulating macrophages and immune cells in general. While it has been described that the redox status of single cysteines in specific proteins is relevant for macrophage functions, global oxidation information is scarce. Hence, we globally assessed the impact of oxidation on macrophage activation using untargeted proteomics and PTM-omics. We exposed THP-1 macrophages to lipopolysaccharide (LPS) for 4 h and 24 h and applied a sequential iodoTMT labeling approach to get information on overall oxidation as well as reversible oxidation of cysteines. Thus, we identified 10452 oxidation sites, which were integratively analyzed with 5057 proteins and 7148 phosphorylation sites to investigate their co-occurance with other omics layers. Based on this integrative analysis, we found significant upregulation of several immune-related pathways, e.g. toll-like receptor 4 (TLR4) signaling, for which 19 proteins, 7 phosphorylation sites, and 39 oxidation sites were significantly affected, highlighting the relevance of oxidations in TLR4-induced macrophage activation. Co-regulation of oxidation and phosphorylation was observed, as evidenced by multiply modified proteins related to inflammatory pathways. Additionally, we observed time-dependent effects, with differences in the dynamics of oxidation sites compared to proteins and phosphorylation sites. Overall, this study highlights the importance of oxidation in regulating inflammatory processes and provides a method that can be readily applied to study the cellular redoxome globally.

## 1 Background

Innate immune cells, such as macrophages, build the first barrier in the defense against foreign substances and pathogens. Complex molecular signaling cascades are triggered upon contact of macrophages with these stimuli. A very prominent example is the pathogen-associated molecular pattern (PAMP) lipopolysaccharide (LPS) from gram-negative bacteria, which is known to induce toll-like receptor 4 (TLR4) signaling (1) with subsequent metabolic reprogramming from oxidative phosphorylation to aerobic glycolysis (2, 3), similar to the Warburg effect in tumors (4).

The involvement of post-translational modifications (PTMs), such as phosphorylation and ubiquitination in innate immunity in general (5) and LPS-induced signaling in macrophages in particular (6), have been described in the past. However, the role of protein oxidation in the regulation of these processes has received only little attention so far, even though it is known that oxidative stress can induce inflammation and vice versa, mediated by the ubiquitous redox-active peroxiredoxin 2 (PRDX2) (7). Furthermore, LPS stimulation of immune cells can induce an oxidative burst, depending on the presence of TLR4, which can be blocked by antioxidants (8). Additionally, the reducing agent glutathione has been shown to affect the production of LPS-induced cytokines (9), highlighting the relevance of the redox status for inflammatory processes.

While almost no global information on redox-regulated proteins is available, referred to as redoxome, several proteins are known to be affected by oxidants and antioxidants modifying specific sites. For example, the oxidation states of cysteines on the ectodomain of TLR2 and TLR4 are critical for LPS-induced signaling (10). For the downstream nuclear factor kappa-light-chain-enhancer of activated B cells (NFκB) it has been described that oxidants enhance its nuclear translocation, while a cysteine in the DNA binding region of its p50 subunit (NFKB1_C62) must be reduced for proper DNA binding once in the nucleus (11–13).

Recently, one preprint and one original article investigated the redoxome of macrophages during activation with LPS. Yan et al., 2023 (14) studied murine immortalized bone marrow-derived macrophages (iBMDM) treated with LPS and Interferon-γ (INFγ) for 24 h and evaluated protein localization-dependent oxidative modifications, focusing on the totality of cysteine oxidation by labeling and quantification of all reduced cysteines. However, they did not consider that cysteine oxidation can occur reversibly or irreversibly, although this is essential information since reversible and irreversible protein oxidation can have different effects. While reversible oxidation is critical for the dynamic regulation of protein structure and activity, irreversible oxidation, mainly occurring under oxidative stress, is most likely associated with a loss of function (15–17). In contrast, the second study, conducted by Hariri et al., 2023 (18), investigated reversible cysteine oxidation after 4 h LPS stimulation of human THP-1 macrophages and found that oxidations are highly important to allow macrophages to respond and adapt to redox and inflammatory challenges efficiently.

Combining the best of both studies, we investigated the effects of LPS on the redoxome of human THP-1 macrophages after 4 h and 24 h. Starting with 20 µg protein in contrast to the previously used ≥500 µg protein, we quantified overall and reversible protein oxidation, applying a sequential iodoTMT labeling approach. By integrating the data with phosphoproteomics data, we aimed to unravel potential co-regulation of these two PTMs. The corresponding full proteome was not only used to correct the changes in PTMs for changes on protein level, which was not done in the previous studies, but was also included in the integrative analysis, revealing detailed insights into the molecular mechanisms induced by LPS in THP-1 macrophages, with the contribution of protein abundance, oxidation, and phosphorylation.

## 2 Methods

**Figure 1:**
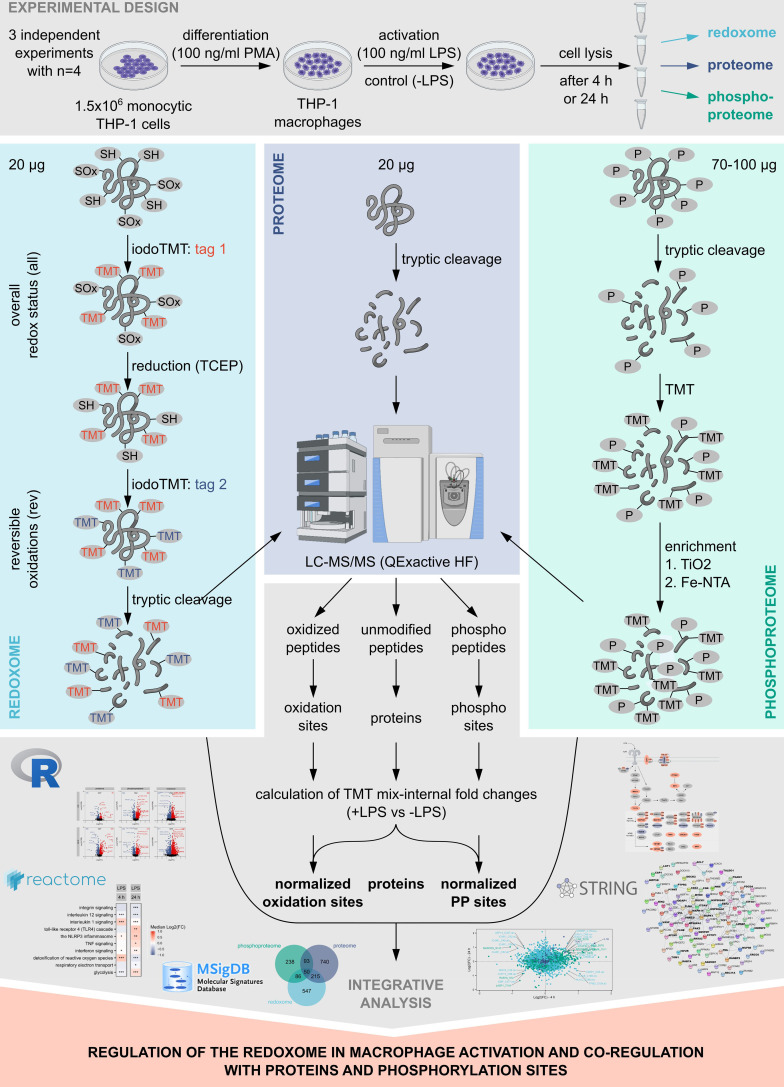
Experimental design. Monocytic THP-1 cells were differentiated to THP-1 macrophages by incubation with PMA for 48 h, followed by a resting period of 24 h. Afterwards, cells were exposed to LPS for 4 h or 24 h, respectively. Cells not treated with LPS served as controls. Proteome, redoxome, and phosphoproteome were created from proteins of the same samples. For the redoxome, a sequential iodoTMT labeling approach was applied to quantify overall (all) and reversible (rev) cysteine oxidation. For the identification of phosphorylated peptides, a two-step enrichment was performed. Intensities obtained for oxidation and phosphorylation sites were normalized to changes in protein level. Finally, changes in protein abundances, oxidation sites, and phosphorylation sites were evaluated integratively to unravel the relevance of the redoxome in macrophage activation and its co-regulation with proteins and phosphorylation sites. Created with BioRender.com.

The applied experimental setup is visualized in **Figure 1**.

### 2.1 Cell culture and treatment

Cell culture and treatment were performed as described before (19). In brief, per sample, 1.5x10^6^ cells of the human monocytic leukemia cell line THP-1 were cultured at 5% CO_2_, 37°C, and 95% humidity in a growth medium consisting of RPMI 1640 medium (GibcoTM, Thermo Fisher Scientific, Waltham, MA, USA), containing 10% fetal bovine serum (FBS, Biowest, Nuaillé, France) and 1% penicillin/streptomycin (Sigma Aldrich, Darmstadt, Germany). They were differentiated to pro-inflammatory macrophages by treatment with 100 ng/ml phorbol-12-myristate-13-acetate (PMA, Sigma Aldrich) for 48 h, followed by 24 h resting in growth medium without PMA. Afterwards, they were exposed to 100 ng/ml lipopolysaccharide (LPS) for 4 h or 24 h. Not LPS-treated cells from the two time points served as controls. After these incubations, cells were washed, harvested and lysed using 150 mM NaCl, 1% Triton X-100, 50 mM Tris HCl pH 7.4, 0.5% sodium deoxycholate, and 0.1% sodium dodecyl sulfate in water, supplemented with protease inhibitor (cOmplete, Roche, Sigma Aldrich) and phosphatase inhibitor (Thermo Scientific™ Halt™ Protease and Phosphatase Inhibitor Cocktail). Lysates were incubated for 60 min on ice and centrifuged for 15 min at 4°C and 16,000 g. According to the manufacturer’s instructions, protein concentrations were determined using the DC protein assay (Bio-Rad, Feldkirchen, Germany).

Three runs of four replicates each were performed, resulting in 12 replicates that were processed equally. Proteome, redoxome, and phosphoproteome were created from the same samples.

### 2.2 Proteome

20 µg protein per sample were prepared for untargeted proteomics using a paramagnetic bead approach (20–22) in combination with enzymatic cleavage using trypsin and tandem mass tag (TMT, TMT-6plex, Thermo Scientific, USA) labeling (see **Supplementary Methods**) as described before (23). The only difference was that the samples were not acidified before loading on the beads for protein clean-up. After labeling, the samples were combined replicate-wise, leading to mixes containing one sample per condition (4 h -LPS, 4 h +LPS, 24 h -LPS, 24 h +LPS). After peptide clean-up, peptides were eluted in two steps, first with 87% ACN in 10 mM ammonium formate (pH 10, Sigma Aldrich), then with 2% dimethylsulfoxide (DMSO, Sigma Aldrich), resulting in two fractions, which were analyzed using liquid chromatography (LC) coupled to a mass spectrometer (MS). In detail, the peptides were separated on a nano-UPLC system (Ultimate 3000, Dionex, USA) with a trapping column (flow rate 5 µl/min, Acclaim PepMap 100 C18, 3 µm, nanoViper, 75 µm × 5 cm, Thermo Fisher, Germany) and an analytical column (flow rate 0.3 µl/min, Acclaim PepMap 100 C18, 3 µm, nanoViper, 75 µm × 25 cm, Thermo Fisher, Germany) using a 160 min non-linear gradient. The nano-UPLC system was coupled to the MS (QExactive HF, Thermo Scientific, USA) via a chip-based ESI source (Nanomate, Advion, USA). According to the previously described workflow (23) precursors between 350 *m/z* and 1550 *m/z* were detected at a resolution of 120K. MS1 AGC target was set to 3e6, with a maximum injection time of 120 ms. The top 15 precursors were isolated using a window of 0.7 Th, with MS2 AGC target 2e5 and a maximum injection time of 120 ms. NCE was 34, fixed first mass 120 *m/z*, and MS2 resolution 60K. A dynamic exclusion of 45 s was used. Further details are provided in the **Supplementary Methods**.

The obtained raw data were processed against the UniProtKB (24) reference proteome of *Homo sapiens* (4 March 2022), using Proteome Discoverer 2.5 and the following parameters: up to 2 missed cleavages, carbamidomethylation (C) and TMT (K) as fixed modifications, oxidation (M), acetylation of the protein N-terminus, and TMT labeling of the peptide N-terminus as variable modifications, correction of reporter ion intensities according to the correction factors provided by the manufacturer. Further details are provided in the Supplementary Methods. This workflow resulted in information on 5057 proteins.

### 2.3 Redoxome

20 µg protein per samples were used for sequential iodoTMT labeling (iodoTMT-6plex, Thermo Scientific, USA). Protein sample volumes were adjusted to 50 µl using 100 mM tetraethylammonium tetrahydroborate (TEAB, Sigma Aldrich, USA). For initial labeling of free thiols (TMT1, reflecting overall oxidation, short: all), two of the available six iodoTMT labels were dissolved in 85 µl methanol each, and 10 µl of the dissolved label was added to the samples, followed by incubation for 1 h protected from light with shaking at 37°C. Afterwards, acetone precipitation was performed by adding six volumes of acetone to the samples and incubating them overnight at -20°C. The next day, samples were centrifuged at 10,000 g and 4°C for 10 min, supernatants were discarded, and air-dried pellets were dissolved in 50 µl 100 mM TEAB. Next, oxidized cysteines were reduced using 5 µl of 50 mM tris(2-carboxyethyl)phosphine hydrochloride (TCEP, Sigma-Aldrich, USA) in 100 mM TEAB and incubation for 1 h with shaking at 50°C. To label the subsequently reduced thiols (TMT2, reflecting reversible oxidation, short: rev), two of the remaining labels were dissolved in 43 µl methanol each, and 5 µl of the dissolved label was added to the samples. After incubation for 1 h protected from light and shaking at 37°C, 2.5 µl 500 mM dithiothreitol (DTT, Sigma Aldrich, USA) in 100 mM TEAB was added to quench the labeling. Samples were combined time point-wise (4 h/24 h: TMT1 -LPS, TMT2 -LPS, TMT1 +LPS, TMT2 +LPS), and another acetone precipitation was performed for 4 h. For proteolytic cleavage of the labeled proteins, 0.4 µg trypsin in 10 µl 100 mM TEAB was added and incubated overnight at 37°C. Desalting was performed using the peptide clean-up on paramagnetic beads as described before (23). For details, see **Supplementary Methods**. In brief, 3 µl beads per sample were prepared by washing them three times with 300 µl water. 250 µl acetonitrile (ACN) was added to the samples before transfer to the beads. After incubation for 8 min at room temperature off the magnetic rack and 2 min incubation on the magnet, supernatants were discarded, and samples were washed with 200 µl 100% ACN. Peptide elution was performed as described for the proteome, resulting in two fractions, which were analyzed on the same LC-MS/MS system as the proteomics samples, using the same parameters.

The obtained raw data were processed against the same UniProtKB (24) reference proteome as the proteome, using Proteome Discoverer 2.5 and the following parameters: up to 2 missed cleavages, iodoTMT as fixed modification (C), oxidation (M) and acetylation of the protein N-terminus as variable modifications, correction of reporter ion intensities according to the correction factors provided by the manufacturer. Further details are provided in the **Supplementary Methods**. This workflow resulted in information on 6576 proteins and 76310 peptide isoforms. Corrected reporter ion intensities of peptide isoforms were translated to site intensities summing the intensities of the peptide isoforms containing the site. The identified peptide isoforms referred to 10452 oxidation sites.

### 2.4 Phosphoproteome

For the phosphoproteome, 70-100 µg protein were used, followed by protein clean-up, proteolytic cleavage with trypsin, TMT labeling (TMTpro-16plex, Thermo Scientific, USA), the combination of all samples from one run (4 times -LPS and +LPS after 4 h and 24 h, all having the same protein amount) and peptide clean-up as described for the proteome. Only the elution after the peptide clean-up was done differently than for proteome and redoxome, not in two fractions but only with water. Afterwards, a two-step enrichment using a workflow based on the HighSelect™ TiO2 Phosphopeptide Enrichment Kit (Thermo Scientific, USA) and the High-Select™ Fe-NTA Phosphopeptide Enrichment Kit (Thermo Scientific, USA) was performed as described before (25).

Obtained samples were analyzed on the same LC-MS/MS system as the proteome and the redoxome, also using a 160 min non-linear gradient but different MS parameters: precursors between 350 *m/z* and 1550 *m/z* were detected at a resolution of 120K. MS1 AGC target was set to 3e6, with a maximum injection time of 150 ms. The top 15 precursors were isolated using a window of 0.7 Th, with MS2 AGC target 2e5 and a maximum injection time 150 ms. NCE was 34, fixed first mass 120 *m/z*, and MS2 resolution 60K. A dynamic exclusion of 45 s was used. For details, see **Supplementary Methods**.

The obtained raw data were processed against the same UniProtKB (24) reference proteome as the proteome and the redoxome, using Proteome Discoverer 2.5 and the following parameters: up to 2 missed cleavages, carbamidomethylation (C) and TMT (K) as fixed modifications, phosphorylation (S/T/Y), oxidation (M), acetylation of the protein N-terminus, and TMT labeling of the peptide N-terminus as variable modifications, correction of reporter ion intensities according to the correction factors provided by the manufacturer. Further details are provided in the **Supplementary Methods**. This workflow resulted in information on 5040 proteins and 42159 peptide isoforms. Corrected reporter ion intensities of peptide isoforms were translated to site intensities summing the intensities of the peptide isoforms containing the site. The identified peptide isoforms referred to 7148 phosphorylation sites, including 5976 on serine, 1127 on threonine, and 45 on tyrosine.

### 2.5 Identification of significantly affected proteins/sites and enrichment analysis

TMT labeling was applied to yield data on all three omics layers. Using this approach, LPS-treated samples (+LPS) of one replicate always became part of one TMT mix with the corresponding not LPS-treated samples (-LPS) from the same biological replicate. Accordingly, TMT mix-internal fold changes (FCs) of +LPS vs -LPS were calculated, resulting in FC data for 12 replicates, which were used for further analyses. This procedure was applied time-point-wise (4 h +LPS vs 4 h -LPS, 24 h +LPS vs 24 h -LPS).

Next, data were filtered for those identified at least in triplicate, resulting in a subset of reliably identified proteins and sites, followed by log2-transformation and median-normalization. Average Log2(FCs) were calculated, and significantly affected proteins and sites were determined using a Student’s t-test based on the replicate Log2(FCs) against 0. Obtained p-values were adjusted for multiple testing, according to Benjamini & Hochberg. Proteins and sites were considered significantly affected with FDR ≤ 0.05.

For the redoxome, the reporter ion intensities referring to overall oxidation (TMT1) reflected reduction levels so far, while the intensities referring to reversible oxidation (TMT2) reflected oxidation. To achieve comparability of the two data sets, Log2(FCs) obtained for the overall oxidation data set (TMT1) were inverted, resulting in positive Log2(FCs) reflecting increased oxidation.

The average Log2(FCs) and FDRs for both time points and all omics layers are summarized in the Additional file.

Enrichment analyses were conducted with significantly affected (FDR ≤ 0.05) proteins/sites using the Reactome (26) gene sets of the MSigDB (27, 28). Enrichment p-values were adjusted for multiple testing, according to Benjamini & Hochberg. Pathways were considered significantly enriched with FDR ≤ 0.05. Direction of regulation was determined based on the median Log2(FC) of all proteins and sites assigned to the pathway. All enriched terms are summarized in the Additional file. Compartments were mapped using the Gene Ontology gene sets of cellular components (29, 30) of the MSigDB (27, 28).

These analyses and the related visualizations were performed in R-3.6.1, using the packages plyr (31), reshape2 (32), xlsx (33), calibrate (34), readxl (35), qpcR (36), splitstackshape (37), tidyr (38), Tmisc (39), ggplot2 (40), circlize (41), ggsci (42), dendsort (43), dendextend (44), biomaRt (45), and msigdbr (46).

Proteins with significantly altered (FDR ≤ 0.05) oxidation and phosphorylation sites after 24 h LPS treatment were uploaded to the STRING-db (47) and visualized using Cytoscape v3.7.2 (48) as well as Cytoscape’s StringApp (49).

## 3 Results

To evaluate the role of cysteine oxidation in macrophage activation and potential co-regulation of protein abundance and phosphorylation, we exposed human THP-1 macrophages to LPS for 4 h and 24 h (**Figure 1**). For the redoxome, a sequential iodoTMT labeling approach was applied to obtain information on overall and reversible cysteine oxidation. The phosphoproteome was generated using a two-step enrichment of phosphopeptides (25). Obtained oxidation and phosphorylation site intensities were normalized to changes on the protein level, which was only possible for modified proteins also part of the proteome (**Figure S1**). This workflow revealed reliable quantification data for 4428 proteins, 4657 overall oxidations, 4355 reversible oxidations, and 2482 phosphorylation sites showing good reproducibility (**Figure S2**) and being assigned to different compartments (**Figure S3**). Significant changes were assessed for all these reliably quantified proteins/sites relative to control samples not treated with LPS.

**Figure 2:**
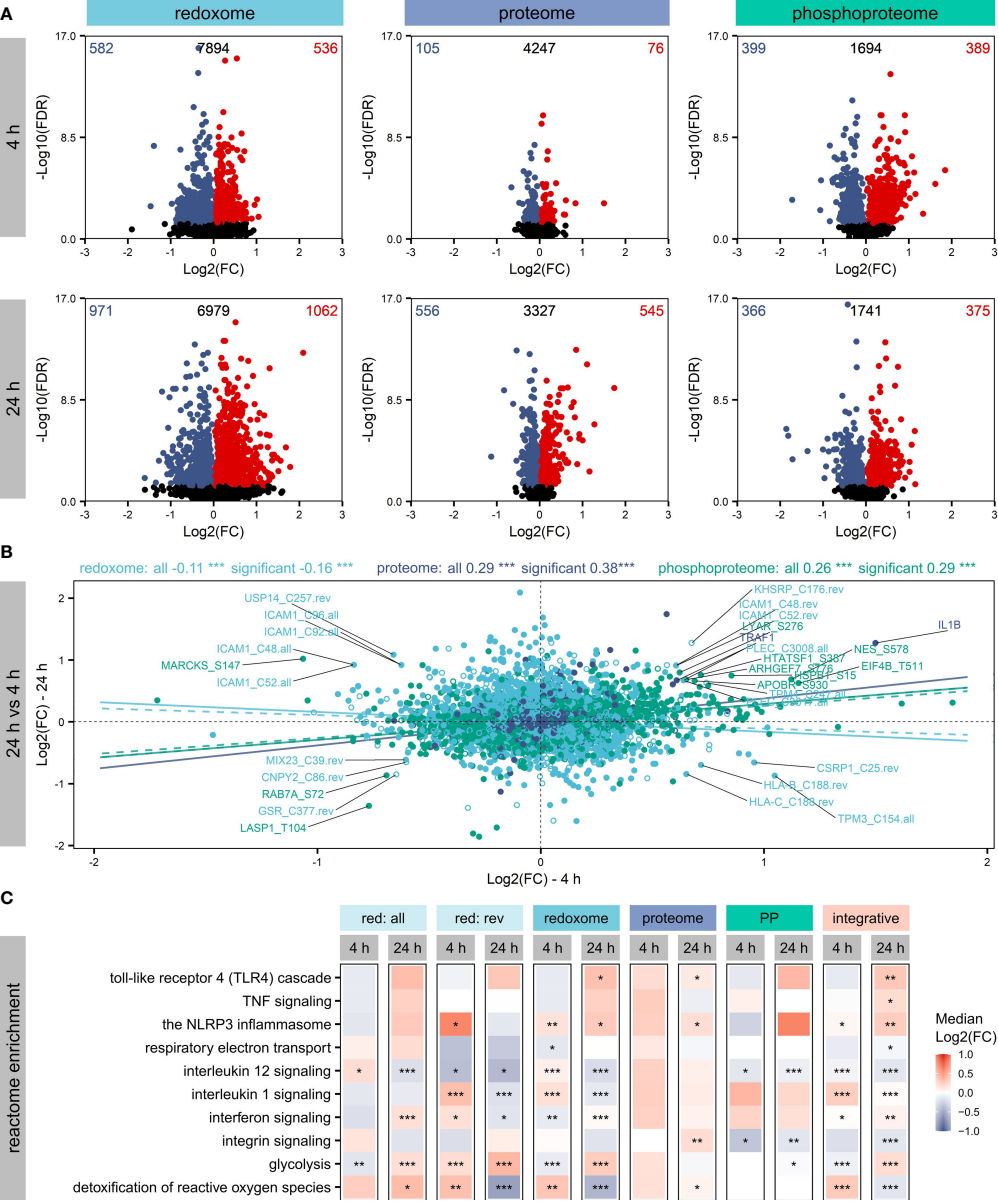
Affected proteins and modification sites. Shown are significantly (FDR ≤ 0.05) altered proteins, phosphorylation sites and oxidation sites after LPS treatment of THP-1 macrophages for 4 h or 24 h, respectively. Log2(FCs) and -Log10(FDRs) are depicted, highlighting the numbers of significantly increased (FDR ≤ 0.05, Log2(FC)>0) or decreased (FDR ≤ 0.05, Log2(FC)<0) proteins/sites in the corners **(A)**. Furthermore, changes in protein/site Log2(FCs) were compared between 4 h and 24 h, distinguishing not affected proteins/sites (empty circles) and significantly altered proteins/sites (filled circles). Pearson correlation coefficients and corresponding significances of correlation (***p-value ≤ 0.001) were determined to get information on the trend of the dynamics within the omics layers. Trendlines reflecting correlation values were added, distinguishing not affected proteins/sites (dashed) and significantly altered proteins/sites (solid) **(B)**. Significantly altered proteins/sites were subjected to an integrative pathway enrichment using the Reactome gene sets provided by the MSigDB. Selected Reactome pathways found significantly (FDR ≤ 0.05) enriched are shown **(C)**. The significance of enrichment is provided with asterisks: *FDR ≤ 0.05, **FDR ≤ 0.01, ***FDR ≤ 0.001. Color reflects the median Log2(FC) of proteins/sites assigned to the pathway.

While all omics layers revealed significantly (FDR ≤ 0.05) altered proteins/sites (**Figure 2A**), most effects were observed for the redoxome, which showed effects on overall (all) and reversible (rev) oxidation, with a higher number of affected sites reflecting overall oxidation (**Figure S4**). Next, we compared proteins bearing significantly affected oxidations with proteins known to be modified on cysteine according to the UniProtKB. Thus, we found proteins known to be modified on cysteine and proteins not described to be oxidized on cysteine so far (**Figure S5**). Examples of significantly altered candidates with known cysteine modification, relevant for inflammatory processes and related redox regulation, are thioredoxin (TXN), glyceraldehyde-3-phosphate dehydrogenase (GAPDH), and Ras-related C3 botulinum toxin substrate 1 (RAC1). Furthermore, the analysis of the Log2(FC) distributions of the significantly affected proteins/sites revealed compartment-dependent trends, which were also time-dependent. For example, many nuclear proteins significantly decreased after 4 h but increased after 24 h. In contrast, the redoxome did not indicate time-dependent modification of nuclear proteins but of proteins from the cytoplasmic region, with significantly increased oxidation mainly after 24 h, accompanied by significant decreases in phosphorylation (**Figure S6**).

To evaluate how the different omics layers are regulated over time, we compared changes after 4 h and 24 h (**Figure 2B**) and found a significant positive correlation for the proteome and the phosphoproteome. In contrast, the redoxome showed the opposite correlation, suggesting a different dynamic of the redoxome.

Next, all significantly altered proteins/sites were subjected to Reactome pathway enrichment to evaluate effects of the PTMs on signaling pathways. Assessment of the top 5 significantly enriched (FDR ≤ 0.05) pathways revealed no significant enrichment of pathways after 4 h based on the proteome (**Figure S7**). In contrast, the PTM layers showed significant enrichment after 4 h and 24 h (**Figure S7**), in line with the observation that PTMs are regulated rather quickly (50). After 24 h, TLR4 signaling, which is known to be induced by LPS (1), was significantly enriched based on significantly altered proteins, oxidation sites (overall and reversible together), and the integrative enrichment using the combination of all omics layers investigated here (**Figure 2C**). Further immune-relevant pathways found significantly upregulated after 24 h based on the integrative analysis were NLRP3 inflammasome, TNF signaling, interferon signaling, and interleukin 1 signaling (**Figure 2C**). In contrast, interleukin 12 signaling and integrin signaling were downregulated. Also, metabolic pathways such as glycolysis, respiratory electron transport, and the detoxification of reactive oxygen species were affected (**Figure 2C**). Comparing the regulation direction after 4 h and 24 h based on the integrative enrichment analysis (**Figure 2C**), we found opposite trends for several pathways, indicating again considerable differences in the regulation at the two time points investigated here.

**Figure 3:**
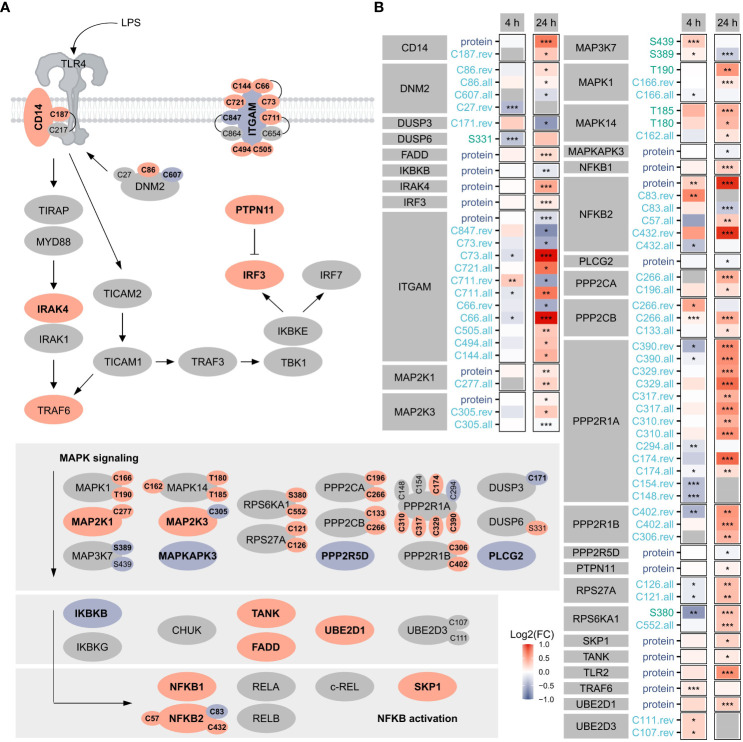
TLR4 signaling. Shown are proteins, phosphorylation sites and oxidation sites assigned to Reactome’s TLR4 signaling after 24 h Significant (FDR ≤ 0.05) changes are depicted in bold. The color reflects the direction of regulation (red: Log2(FC)>0, blue: Log2(FC)<0). Oxidation sites involved in disulfide bonds (according to the UniProtKB) are connected **(A)**. Furthermore, changes in proteins and modification sites assigned to Reactome’s TLR4 signaling were compared after 4 h and 24 h, where the color reflects the direction of the change and the significance is provided with asterisks: *FDR ≤ 0.05, **FDR ≤ 0.01, ***FDR ≤ 0.001 **(B)**.

Focusing on TLR4 signaling as a benchmark pathway (**Figure 3**) known to be induced by LPS treatment of macrophages (1), we found 19 proteins, 7 phosphorylation sites, and 39 oxidation sites (overall, reversible, or both) related to this pathway significantly affected after 4 h or 24 h, highlighting the importance of oxidation in the regulation downstream of TLR4. Notably, more proteins/sites were significantly altered after 24 h, suggesting the involvement of PTMs also at later time points. Furthermore, opposite changes were induced for many modification sites after 4 h and 24 h.

For the affected TLR4 signaling-related oxidation sites identified here, cysteines involved in disulfide bonds were identified using the UniProtKB (**Figure 3A**), revealing most of those not known to be modified so far.

**Figure 4:**
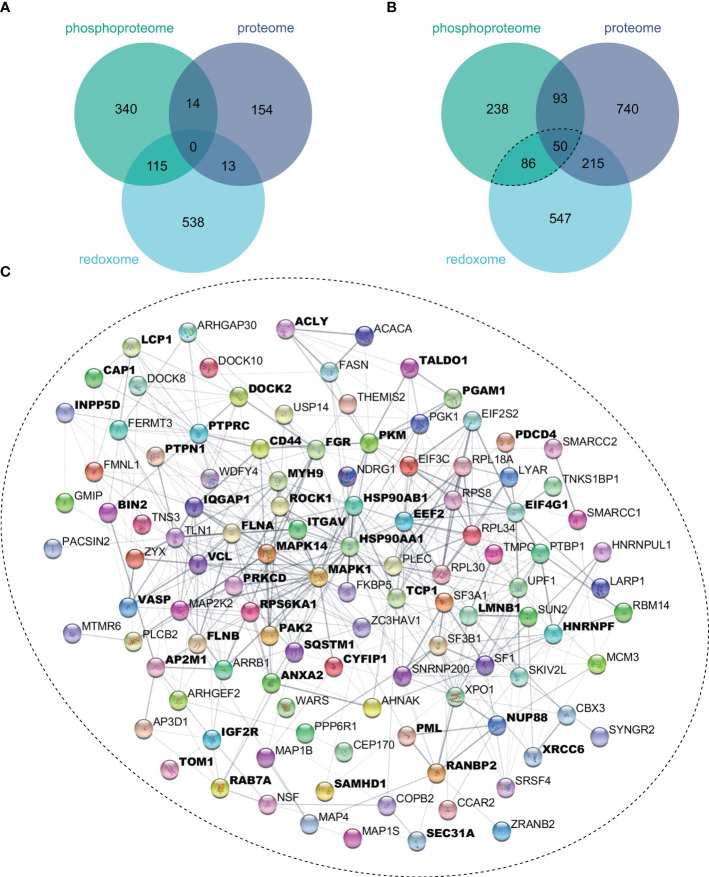
Multiply modified proteins. Proteins showing significant (FDR ≤ 0.05) changes in protein level, phosphorylation sites, or oxidation sites after 4 h **(A)** or 24 h **(B)** of LPS treatment were compared. Proteins with significantly affected phosphorylation and oxidation sites were subjected to the String-db, where an enrichment analysis revealed significant enrichment (FDR ≤ 0.05) of Reactome’s inflammatory processes, for which all assigned proteins were marked bold **(C)**.

To evaluate the extent to which oxidation and phosphorylation regulate inflammatory processes, proteins with significantly altered oxidation or phosphorylation sites after 4 h (**Figure 4A**) or 24 h (**Figure 4B**) LPS stimulation were determined. Thus, we found more proteins to be multiply modified after 24 h, which were subjected to the STRING-db (47), and proteins related to inflammatory processes were identified using Cytoscape’s StringApp (49). Enrichment analysis revealed that several of these multiply modified proteins are related to inflammatory processes (**Figure 4C**), highlighting the importance of oxidation and phosphorylation in regulating inflammatory processes in THP-1 macrophages.

## 4 Discussion

Since global information on the impact of cysteine oxidations in inflammatory processes is scarce, we assessed overall and reversible cysteine oxidation and investigated whether it co-occurs with phosphorylation, being one of the most extensively studied PTMs.

To quantify cysteine oxidation, we applied an optimized sequential iodoTMT labeling approach, starting with only 20 µg protein, which is less than used before in macrophages using different techniques (14, 18) or in other contexts using iodoTMT (51, 52). This approach resulted in 10452 oxidation sites, comparable to or more than identified in these previous studies.

Comparing those to proteins already described to be oxidized on cysteine and showing significant effects on cysteine modification here, we found TXN, GAPDH, and RAC1, for instance. TXN is responsible for the de-nitrosylation of proteins, thus influencing inflammatory processes (53). According to the UniProtKB, TXN has a redox-reactive disulfide bond (C32-C35) and three S-nitrosocysteines (C62, C69, C73) (54). Notably, we identified all these modification sites, and the peptide containing C62 and C69 showed significantly decreased reversible oxidation, which was not observed in overall oxidation. GAPDH is also involved in the processes induced by macrophage activation, where its S-nitrosylation supports the induction of apoptosis (55). According to the UniProtKB, its cysteines C152 (predicted by similarity) and C247 (56) are known for their potential to be modified. Additionally, we got information on C156 with the approach described here. However, this site was on the same peptide as C152. Thus, the significant increase of this peptide could occur due to either of the two cysteines. RAC1, which has been described to be induced by LPS in macrophages, subsequently leading to ROS formation and NFκB-dependent production of pro-inflammatory cytokines (57), can be modified on C189 (58) according to the UniProtKB. While we were not able to get information on this site, we got quantitation data on the sites C6, C105, C157, and C178, of which C105, C157, and C178 were found to be significantly affected, either based on overall oxidation or reversible oxidation, confirming the importance of assessing overall as well as reversible oxidation.

Based on all significantly altered oxidation sites, identified proteins, and phosphorylation sites, we found regulation of typical LPS-induced pathways like TLR4 signaling. Having a closer look at the proteins and sites assigned to this pathway, we discovered that oxidation was regulating this pathway to a higher extent than phosphorylation, although it must be noted that a two-step enrichment with TiO2 and Fe-NTA columns was used to examine changes in phosphorylation sites. Unfortunately, this approach is ineffective in enriching phosphorylated tyrosine, a main driver of TLR signaling (59), requiring implementing antibody-based tyrosin phosphorylation strategies in future studies.

For LPS-activated macrophages, metabolic reprogramming from oxidative phosphorylation to glycolysis is described (2, 3). While studies are available noticing this also in THP-1 macrophages (60), this reprogramming may differ between humans and mice (61). Here, we observed a time-dependent regulation of glycolysis and respiratory electron transport chain, mainly driven by cysteine oxidation, highlighting the importance of this PTM.

It is known that different combinations of PTMs can induce different processes, even though the biological outcomes are only beginning to be understood (62). For example, the NLRP3 inflammasome is regulated by phosphorylation, ubiquitination, sumolyation, and S-nitrosylation (63, 64). Thus, we investigated proteins bearing significantly affected phosphorylated and oxidized sites in more detail. We found that many proteins involved in inflammatory processes are regulated by phosphorylation and oxidation, which highlights the importance of considering PTMs when investigating modes of action, of which certainly phosphorylation and oxidation are highly relevant.

Besides the role of protein oxidation in inflammatory processes, this modification is also very relevant for several diseases (65), e.g. cancer, which can be accompanied by the excessive production of ROS (66). In recent years, targeting of disease-related proteins with covalent inhibitors emerged (67), which is using a ligand containing an electrophilic “warhead” that forms a covalent adduct with a nucleophilic residue like cysteine on the protein, ideally with minimal influence on key non-covalent interactions (68, 69). Alternatively, nucleophilic fragments can directly target cysteines oxidized to sulfenic acid, leading to changes in the cellular redox status (70). This approach has been applied to target kinases, for instance, due to their role in various diseases (71, 72). Furthermore, a nucleophilic ligand preferably targeting tyrosine phosphatases has been identified (73), which are known to be regulated by cysteine oxidation (74), further highlighting the relevance of the interplay of oxidation and phosphorylation. Therefore, the approach presented here, identifying relevant oxidation sites and investigating the interplay between cysteine oxidation and phosphorylation, can provide valuable information for drug discovery, potentially revolutionizing the therapy of diseases. In addition, it should be noted that it might be beneficial to complement data on protein oxidation, which can be easily assessed using the approach described here, with information on RNA oxidation, which is also highly relevant in diseases and pathological conditions and has the potential to mediate inflammatory responses (75).

Based on the data presented here, functional analyses should be conducted next to validate the obtained results. However, this study provides comprehensive data underpinning the importance of oxidations in inflammatory processes in general and LPS-driven macrophage activation in particular. Consequently, the redoxome should receive more attention in future. Due to the pronounced time-dependent effects and differences in the dynamics of the investigated omics layers observed here, we recommend generating time-resolved data also in future, which will allow deep mechanistic insights.

## Data availability statement

The raw data have been deposited to the ProteomeXchange Consortium via the PRIDE (76) partner repository with the dataset identifiers PXD043025, PXD043139, and PXD042912, as well as the DOIs 10.6019/PXD043025, 10.6019/PXD043139, and 10.6019/PXD042912 for the proteome, the redoxome, and the phosphoproteome, respectively.

## Ethics statement

Ethical approval was not required for the studies on humans in accordance with the local legislation and institutional requirements because only commercially available established cell lines were used.

## Author contributions

IK, HG, KS, and MB contributed to the conception and design of the study. IK, SF, and HG prepared the samples. IK, SF, and HG analyzed the data. IK prepared the figures. IK wrote the first draft of the manuscript. All authors contributed to the manuscript’s revision, read, and approved the submitted version.

## References

[1] Tsukamoto H Takeuchi S Kubota K Kobayashi Y Kozakai S Ukai I. Lipopolysaccharide (LPS)-binding protein stimulates CD14-dependent Toll-like receptor 4 internalization and LPS-induced TBK1-IKK-IRF3 axis activation. J Biol Chem (2018) 293(26):10186–201. doi: 10.1074/jbc.M117.796631 PMC6028956 29760187

[2] Palsson-McDermott EM O’Neill LAJ. The Warburg effect then and now: From cancer to inflammatory diseases. BioEssays (2013) 35(11):965–73. doi: 10.1002/bies.201300084 24115022

[3] Kelly B O’Neill LAJ. Metabolic reprogramming in macrophages and dendritic cells in innate immunity. Cell Res (2015) 25(7):771–84. doi: 10.1038/cr.2015.68 PMC4493277 26045163

[4] Warburg O. Iron, the oxygen-carrier of respiration-ferment. Science (1925) 61(1588):575–82. doi: 10.1126/science.61.1588.575 17837805

[5] Liu J Qian C Cao X. Post-translational modification control of innate immunity. Immunity (2016) 45(1):15–30. doi: 10.1016/j.immuni.2016.06.020 27438764

[6] Rossol M Heine H Meusch U Quandt D Klein C Sweet MJ. LPS-induced cytokine production in human monocytes and macrophages. Crit Rev Immunol (2011) 31(5):379–446. doi: 10.1615/CritRevImmunol.v31.i5.20 22142165

[7] Salzano S Checconi P Hanschmann EM Lillig CH Bowler LD Chan P. Linkage of inflammation and oxidative stress via release of glutathionylated peroxiredoxin-2, which acts as a danger signal. Proc Natl Acad Sci U S A (2014) 111(33):12157–62. doi: 10.1073/pnas.1401712111 PMC4143057 25097261

[8] Ryan KA Smith MF Jr. Sanders MK Ernst PB. Reactive oxygen and nitrogen species differentially regulate Toll-like receptor 4-mediated activation of NF-kappa B and interleukin-8 expression. Infect Immun (2004) 72(4):2123–30. doi: 10.1128/IAI.72.4.2123-2130.2004 PMC375203 15039334

[9] Dobashi K Aihara M Araki T Shimizu Y Utsugi M Iizuka K. Regulation of LPS induced IL-12 production by IFN-gamma and IL-4 through intracellular glutathione status in human alveolar macrophages. Clin Exp Immunol (2001) 124(2):290–6. doi: 10.1046/j.1365-2249.2001.01535.x PMC1906042 11422207

[10] Zsengellér ZK Gerard NP. The oxidation state of cysteine thiols on the ectodomain of TLR2 and TLR4 influences intracellular signaling. Immunobiol (2020) 225(2):151895. doi: 10.1016/j.imbio.2019.12.004 31843260

[11] Toledano MB Leonard WJ. Modulation of transcription factor NF-kappa B binding activity by oxidation-reduction in vitro. Proc Natl Acad Sci (1991) 88(10):4328–32. doi: 10.1073/pnas.88.10.4328 PMC51652 1903539

[12] Nishi T Shimizu N Hiramoto M Sato I Yamaguchi Y Hasegawa M. Spatial redox regulation of a critical cysteine residue of NF-κB in vivo. J Biol Chem (2002) 277(46):44548–56. doi: 10.1074/jbc.M202970200 12213807

[13] Mohora M Greabu M Totan A Mitrea N Battino M. Redox-sensitive signaling factors and antioxidants. Farmacia (2009) 57(4):399–410.

[14] Yan T Julio AR Villanueva M Jones AE Ball AB Boatner LM. Proximity-labeling chemoproteomics defines the subcellular cysteinome and inflammation-responsive mitochondrial redoxome. Cell Chem Biol (2023) 30(7):811–27.e7. doi: 10.1016/j.chembiol.2023.06.008 PMC10510412 37419112

[15] Mannaa A Hanisch FG. Redox proteomes in human physiology and disease mechanisms. J Proteome Res (2019) 19(1):1–17. doi: 10.1021/acs.jproteome.9b00586 31647248

[16] Giustarini D Milzani A Aldini G Carini M Rossi R Dalle-Donne I. S-nitrosation versus S-glutathionylation of protein sulfhydryl groups by S-nitrosoglutathione. Antioxid Redox Signal (2005) 7(7-8):930–9. doi: 10.1089/ars.2005.7.930 15998248

[17] Dalle-Donne I Scaloni A Giustarini D Cavarra E Tell G Lungarella G. Proteins as biomarkers of oxidative/nitrosative stress in diseases: The contribution of redox proteomics. Mass Spectrometry Rev (2005) 24(1):55–99. doi: 10.1002/mas.20006 15389864

[18] Abu Hariri H Braunstein I Salti T Glaser F Gefen T Geva-Zatorsky N. Global thiol proteome analysis provides novel insights into the macrophage inflammatory response and its regulation by the thioredoxin system. Antioxid Redox Signal (2023) 38(4-6):388–402. doi: 10.1089/ars.2022.0026 35979894

[19] Schaffert A Arnold J Karkossa I Blüher M von Bergen M Schubert K. The emerging plasticizer alternative DINCH and its metabolite MINCH induce oxidative stress and enhance inflammatory responses in human THP-1 macrophages. Cells (2021) 10(9):2367. doi: 10.3390/cells10092367 34572016 PMC8466537

[20] Hughes CS Foehr S Garfield DA Furlong EE Steinmetz LM Krijgsveld J. Ultrasensitive proteome analysis using paramagnetic bead technology. Mol Syst Biol (2014) 10:757. doi: 10.15252/msb.20145625 25358341 PMC4299378

[21] Hughes CS Moggridge S Muller T Sorensen PH Morin GB Krijgsveld J. Single-pot, solid-phase-enhanced sample preparation for proteomics experiments. Nat Protoc (2019) 14:68–85. doi: 10.1038/s41596-018-0082-x 30464214

[22] Bannuscher A Karkossa I Buhs S Nollau P Kettler K Balas M. A multi-omics approach reveals mechanisms of nanomaterial toxicity and structure–activity relationships in alveolar macrophages. Nanotoxicology (2019) 14(2):181–95. doi: 10.1080/17435390.2019.1684592 31774342

[23] Wang Z Karkossa I Großkopf H Rolle-Kampczyk U Hackermüller J von Bergen M. Comparison of quantitation methods in proteomics to define relevant toxicological information on AhR activation of HepG2 cells by BaP. Toxicology (2021) 448:152652. doi: 10.1016/j.tox.2020.152652 33278487

[24] Consortium TU. UniProt: the universal protein knowledgebase in 2023. Nucleic Acids Res (2022) 51(D1):D523–D31. doi: 10.1093/nar/gkac1052 PMC9825514 36408920

[25] Großkopf H Walter K Karkossa I von Bergen M Schubert K. Non-genomic ahR-signaling modulates the immune response in endotoxin-activated macrophages after activation by the environmental stressor baP. Front Immunol (2021) 12(1008). doi: 10.3389/fimmu.2021.620270 PMC8045971 33868237

[26] Gillespie M Jassal B Stephan R Milacic M Rothfels K Senff-Ribeiro A. The reactome pathway knowledgebase 2022. Nucleic Acids Res (2021) 50(D1):D687–D92. doi: 10.1093/nar/gkab1028 PMC8689983 34788843

[27] Subramanian A Tamayo P Mootha VK Mukherjee S Ebert BL Gillette MA. Gene set enrichment analysis: A knowledge-based approach for interpreting genome-wide expression profiles. Proc Natl Acad Sci (2005) 102(43):15545–50. doi: 10.1073/pnas.0506580102 PMC1239896 16199517

[28] Liberzon A Subramanian A Pinchback R Thorvaldsdóttir H Tamayo P Mesirov JP. Molecular signatures database (MSigDB) 3.0. Bioinformatics (2011) 27(12):1739–40. doi: 10.1093/bioinformatics/btr260 PMC3106198 21546393

[29] Ashburner M Ball CA Blake JA Botstein D Butler H Cherry JM. Gene Ontology: tool for the unification of biology. Nat Genet (2000) 25(1):25–9. doi: 10.1038/75556 PMC3037419 10802651

[30] Consortium TGO Aleksander SA Balhoff J Carbon S Cherry JM Drabkin HJ. The gene ontology knowledgebase in 2023. Genetics (2023) 224(1). doi: 10.1093/genetics/iyad031 PMC10158837 36866529

[31] Wickham H. The split-apply-combine strategy for data analysis. J Stat Softw (2011) 40(1):1–29. doi: 10.18637/jss.v040.i01

[32] Wickham H. Reshaping data with there shape Package. J Stat Softw (2007) 21(12):1–20. doi: 10.18637/jss.v021.i12

[33] Adrian AD Cole A. xlsx: Read, Write, Format Excel 2007 and Excel 97/2000/XP/2003 Files. R package version 0.6.1 (2018). Available at: https://CRAN.R-project.org/package=xlsx.

[34] Jan G. calibrate: Calibration of Scatterplot and Biplot Axes. R package version 1.7.5 (2019). Available at: https://CRAN.R-project.org/package=calibrate.

[35] Hadley W Jennifer B. readxl: Read Excel Files. R package version 1.3.1 (2019). Available at: https://CRAN.R-project.org/package=readxl.

[36] Andrej-Nikolai S. qpcR: Modelling and Analysis of Real-Time PCR Data. R package version 1.4-1 (2018). Available at: https://CRAN.R-project.org/package=qpcR.

[37] Ananda M. splitstackshape: Stack and Reshape Datasets After Splitting Concatenated Values. R package version 1.4.8 (2019). Available at: https://CRAN.R-project.org/package=splitstackshape.

[38] Hadley W Lionel H. tidyr: Tidy Messy Data. R package version 1.0.0 (2019). Available at: https://CRAN.R-project.org/package=tidyr.

[39] Stephen T. Tmisc: Turner Miscellaneous. R package version 0.1.22 (2019). Available at: https://CRAN.R-project.org/package=Tmisc.

[40] Hadley W. ggplot2: Elegant Graphics for Data Analysis. New York: Springer-Verlag (2016).

[41] Gu Z. circlize implements and enhances circular visualization in R. Bioinformatics (2014) 30(19):2811–2. doi: 10.1093/bioinformatics/btu393 24930139

[42] Nan X. ggsci: Scientific Journal and Sci-Fi Themed Color Palettes for ‘ggplot2’. R package version 2.9 (2018). Available at: https://CRAN.R-project.org/package=ggsci.

[43] Sakai R Winand R Verbeiren T Moere AV Aerts J. dendsort: modular leaf ordering methods for dendrogram representations in R. F1000Res (2014) 3:177–. doi: 10.12688/f1000research.4784.1 PMC4162509 25232468

[44] Galili T. dendextend: an R package for visualizing, adjusting and comparing trees of hierarchical clustering. Bioinformatics (2015) 31(22):3718–20. doi: 10.1093/bioinformatics/btv428 PMC4817050 26209431

[45] Durinck S Spellman PT Birney E Huber W. Mapping identifiers for the integration of genomic datasets with the R/Bioconductor package biomaRt. Nat Protoc (2009) 4(8):1184. doi: 10.1038/nprot.2009.97 19617889 PMC3159387

[46] Dolgalev I. msigdbr: MSigDB gene sets for multiple organisms in a tidy data format. (2019).

[47] Szklarczyk D Kirsch R Koutrouli M Nastou K Mehryary F Hachilif R. The STRING database in 2023: protein-protein association networks and functional enrichment analyses for any sequenced genome of interest. Nucleic Acids Res (2023) 51(D1):D638–d46. doi: 10.1093/nar/gkac1000 PMC9825434 36370105

[48] Shannon P Markiel A Ozier O Baliga NS Wang JT Ramage D. Cytoscape: a software environment for integrated models of biomolecular interaction networks. Genome Res (2003) 13(11):2498–504. doi: 10.1101/gr.1239303 PMC403769 14597658

[49] Doncheva NT Morris JH Gorodkin J Jensen LJ. Cytoscape stringApp: network analysis and visualization of proteomics data. J Proteome Res (2019) 18(2):623–32. doi: 10.1021/acs.jproteome.8b00702 PMC6800166 30450911

[50] Canzler S Schor J Busch W Schubert K Rolle-Kampczyk UE Seitz H. Prospects and challenges of multi-omics data integration in toxicology. Arch Toxicol (2020) 94(2):371–88. doi: 10.1007/s00204-020-02656-y 32034435

[51] Wei G Wang C Lei X Gao X Li J Zhang S. IodoTMT-labeled redox proteomics reveals the involvement of oxidative post-translational modification in response to para-hydroxybenzoic acid and hydrogen peroxide stresses in poplar. Ecotoxicol Environ safety (2023) 259:115033. doi: 10.1016/j.ecoenv.2023.115033 37224778

[52] Araki K Kusano H Sasaki N Tanaka R Hatta T Fukui K. Redox sensitivities of global cellular cysteine residues under reductive and oxidative stress. J Proteome Res (2016) 15(8):2548–59. doi: 10.1021/acs.jproteome.6b00087 27350002

[53] Hernansanz-Agustín P Izquierdo-Álvarez A García-Ortiz A Ibiza S Serrador JM Martínez-Ruiz A. Nitrosothiols in the immune system: signaling and protection. Antioxid Redox Signaling (2012) 18(3):288–308. doi: 10.1089/ars.2012.4765 PMC3518543 22746191

[54] Weichsel A Brailey JL Montfort WR. Buried S-nitrosocysteine revealed in crystal structures of human thioredoxin. Biochemistry (2007) 46(5):1219–27. doi: 10.1021/bi061878r 17260951

[55] Hara MR Agrawal N Kim SF Cascio MB Fujimuro M Ozeki Y. S-nitrosylated GAPDH initiates apoptotic cell death by nuclear translocation following Siah1 binding. Nat Cell Biol (2005) 7(7):665–74. doi: 10.1038/ncb1268 15951807

[56] Jia J Arif A Willard B Smith JD Stuehr DJ Hazen SL. Protection of extraribosomal RPL13a by GAPDH and dysregulation by S-nitrosylation. Mol Cell (2012) 47(4):656–63. doi: 10.1016/j.molcel.2012.06.006 PMC3635105 22771119

[57] Sanlioglu S Williams CM Samavati L Butler NS Wang G McCray PB Jr.. Lipopolysaccharide induces rac1-dependent reactive oxygen species formation and coordinates tumor necrosis factor-α Secretion through IKK regulation of NF-κB *. J Biol Chem (2001) 276(32):30188–98. doi: 10.1074/jbc.M102061200 11402028

[58] Kinsella BT Erdman RA Maltese WA. Carboxyl-terminal isoprenylation of ras-related GTP-binding proteins encoded by rac1, rac2, and ralA. J Biol Chem (1991) 266(15):9786–94. doi: 10.1016/S0021-9258(18)92889-9 1903399

[59] Page TH Smolinska M Gillespie J Urbaniak AM Foxwell BMJ. Tyrosine kinases and inflammatory signalling. Curr Mol Med (2009) 9(1):69–85. doi: 10.2174/156652409787314507 19199943

[60] Ubanako P Xelwa N Ntwasa M. LPS induces inflammatory chemokines via TLR-4 signalling and enhances the Warburg Effect in THP-1 cells. PloS One (2019) 14(9):e0222614. doi: 10.1371/journal.pone.0222614 31560702 PMC6764657

[61] Vijayan V Pradhan P Braud L Fuchs HR Gueler F Motterlini R. Human and murine macrophages exhibit differential metabolic responses to lipopolysaccharide - A divergent role for glycolysis. Redox Biol (2019) 22:101147. doi: 10.1016/j.redox.2019.101147 30825774 PMC6396203

[62] Lothrop AP Torres MP Fuchs SM. Deciphering post-translational modification codes. FEBS Lett (2013) 587(8):1247–57. doi: 10.1016/j.febslet.2013.01.047 PMC3888991 23402885

[63] Yang J Liu Z Xiao TS. Post-translational regulation of inflammasomes. Cell Mol Immunol (2017) 14(1):65–79. doi: 10.1038/cmi.2016.29 27345727 PMC5214939

[64] Kelley N Jeltema D Duan Y He Y. The NLRP3 inflammasome: an overview of mechanisms of activation and regulation. Int J Mol Sci (2019) 20(13):3328. doi: 10.3390/ijms20133328 31284572 PMC6651423

[65] Kehm R Baldensperger T Raupbach J Höhn A. Protein oxidation - Formation mechanisms, detection and relevance as biomarkers in human diseases. Redox Biol (2021) 42:101901. doi: 10.1016/j.redox.2021.101901 33744200 PMC8113053

[66] Moloney JN Cotter TG. ROS signalling in the biology of cancer. Semin Cell Dev Biol (2018) 80:50–64. doi: 10.1016/j.semcdb.2017.05.023 28587975

[67] Maurais AJ Weerapana E. Reactive-cysteine profiling for drug discovery. Curr Opin Chem Biol (2019) 50:29–36. doi: 10.1016/j.cbpa.2019.02.010 30897495 PMC6584045

[68] Keeley A Ábrányi-Balogh P Keserű GM. Design and characterization of a heterocyclic electrophilic fragment library for the discovery of cysteine-targeted covalent inhibitors. MedChemComm (2019) 10(2):263–7. doi: 10.1039/C8MD00327K PMC6390469 30881613

[69] Mukherjee H Grimster NP. Beyond cysteine: recent developments in the area of targeted covalent inhibition. Curr Opin Chem Biol (2018) 44:30–8. doi: 10.1016/j.cbpa.2018.05.011 29857316

[70] Fu L Jung Y Tian C Ferreira RB Cheng R He F. Nucleophilic covalent ligand discovery for the cysteine redoxome. Nat Chem Biol (2023). doi: 10.1038/s41589-023-01330-5 PMC12262106 37248412

[71] Liu Q Sabnis Y Zhao Z Zhang T Buhrlage Sara J Jones Lyn H. Developing irreversible inhibitors of the protein kinase cysteinome. Chem Biol (2013) 20(2):146–59. doi: 10.1016/j.chembiol.2012.12.006 PMC3583020 23438744

[72] Lu X Smaill JB Patterson AV Ding K. Discovery of cysteine-targeting covalent protein kinase inhibitors. J Medicinal Chem (2022) 65(1):58–83. doi: 10.1021/acs.jmedchem.1c01719 34962782

[73] Gupta V Yang J Liebler DC Carroll KS. Diverse redoxome reactivity profiles of carbon nucleophiles. J Am Chem Society (2017) 139(15):5588–95. doi: 10.1021/jacs.7b01791 PMC5898444 28355876

[74] Karisch R Fernandez M Taylor P Virtanen C St-Germain Jonathan R Jin Lily L. Global proteomic assessment of the classical protein-tyrosine phosphatome and “Redoxome”. Cell (2011) 146(5):826–40. doi: 10.1016/j.cell.2011.07.020 PMC3176638 21884940

[75] Tanaka M Chock PB. Oxidative modifications of RNA and its potential roles in biosystem. Front Mol Biosci (2021) 8. doi: 10.3389/fmolb.2021.685331 PMC8149912 34055897

[76] Perez-Riverol Y Csordas A Bai J Bernal-Llinares M Hewapathirana S Kundu DJ. The PRIDE database and related tools and resources in 2019: improving support for quantification data. Nucleic Acids Res (2019) 47(D1):D442–d50. doi: 10.1093/nar/gky1106 PMC6323896 30395289

