# Supplementary material for: Oxidation is an underappreciated post-translational modification in the regulation of immune responses associated with changes in phosphorylation
Source: Front Immunol. 2023 Sep 22;14:1244431. doi: 10.3389/fimmu.2023.1244431 (PMC10559879; doi:10.3389/fimmu.2023.1244431)
Supplement: Supplementary file 2 [file DataSheet_2.pdf]

Supplementary Material

Oxidation is an underappreciated post-translational modification in the regulation of immune responses associated with changes in phosphorylation

Isabel Karkossa[1], Sabine Fürst[1], Henning Großkopf[1,2], Martin von Bergen[1,3,4], Kristin Schubert[1*]

[1] Department of Molecular Systems Biology, Helmholtz-Centre for Environmental Research - UFZ, Leipzig, Germany

[2] Present Address: Department of Neurology, Martin-Luther University Halle-Wittenberg, Halle (Saale), Germany

[3] Institute of Biochemistry, Leipzig University, Leipzig, Germany

[4] German Centre for Integrative Biodiversity Research (iDiv) Halle-Jena-Leipzig, Leipzig, Germany

*** Correspondence:**
Kristin Schubert
kristin.schubert@ufz.de

Contents

1 Supplementary Figures 3
2 Supplementary Methods 7
2.1 Proteome 7
2.1.1 Sample preparation on paramagnetic beads 7
2.1.2 LC-MS data acquisition 8
2.1.3 Data processing using Proteome Discoverer 2.5 10
2.2 Redoxome 12
2.2.1 Sample preparation 12
2.2.2 LC-MS data acquisition 12
2.2.3 Data processing using Proteome Discoverer 2.5 13
2.3 Phosphoproteome 15
2.3.1 Sample preparation 15
2.3.2 LC-MS data acquisition 15
2.3.3 Data processing using Proteome Discoverer 2.5 17
3 References 20

Figures

Figure S1: Overlaps in proteome proteins and proteins found phosphorylated or oxidized. 3
Figure S2: Principal component analyses (PCAs) of reliably identified proteins and sites. 3
Figure S3: Assignment of identified proteins to cellular compartments. 4
Figure S4: Affected oxidation sites. 4
Figure S5: Overlap of affected oxidized proteins with proteins known to be modified on cysteine. 5
Figure S6: Compartment densities. 5
Figure S7: Top 5 significantly enriched Reactome pathways. 6

Tables

Table S1: LC gradient proteome. 8
Table S2: MS parameters proteome. 9
Table S3: Processing workflow parameters proteome. 10
Table S4: Consensus workflow general parameters proteome. 11
Table S5: Consensus workflow quantification parameters proteome. 11
Table S6: Processing workflow parameters redoxome. 13
Table S7: Consensus workflow general parameters redoxome. 14
Table S8: Consensus workflow quantification parameters redoxome. 14
Table S9: LC gradient phosphoproteome. 15
Table S10: MS parameters phosphoproteome. 16
Table S11: Processing workflow parameters phosphoproteome. 17
Table S12: Consensus workflow general parameters phosphoproteome. 18
Table S13: Consensus workflow quantification parameters phosphoproteome. 19

1 Supplementary Figures

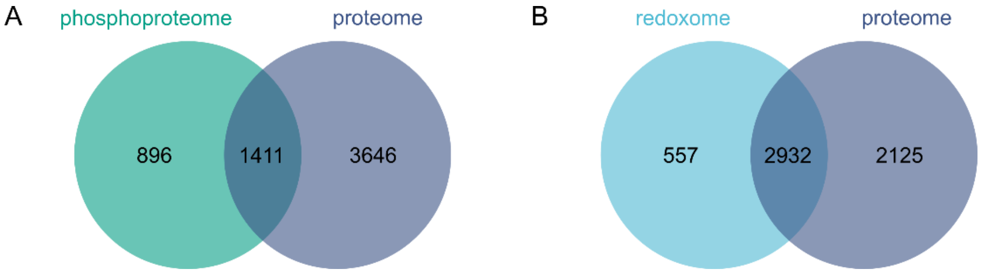

Figure S1: Overlaps in proteome proteins and proteins found phosphorylated or oxidized. Shown are the overlaps of proteins found in the proteome and such for which phosphorylation sites (A) or oxidation sites (B) were identified. Since site intensities were normalized to changes on the protein level, only modified proteins with the protein intensity available in the proteome were investigated further.

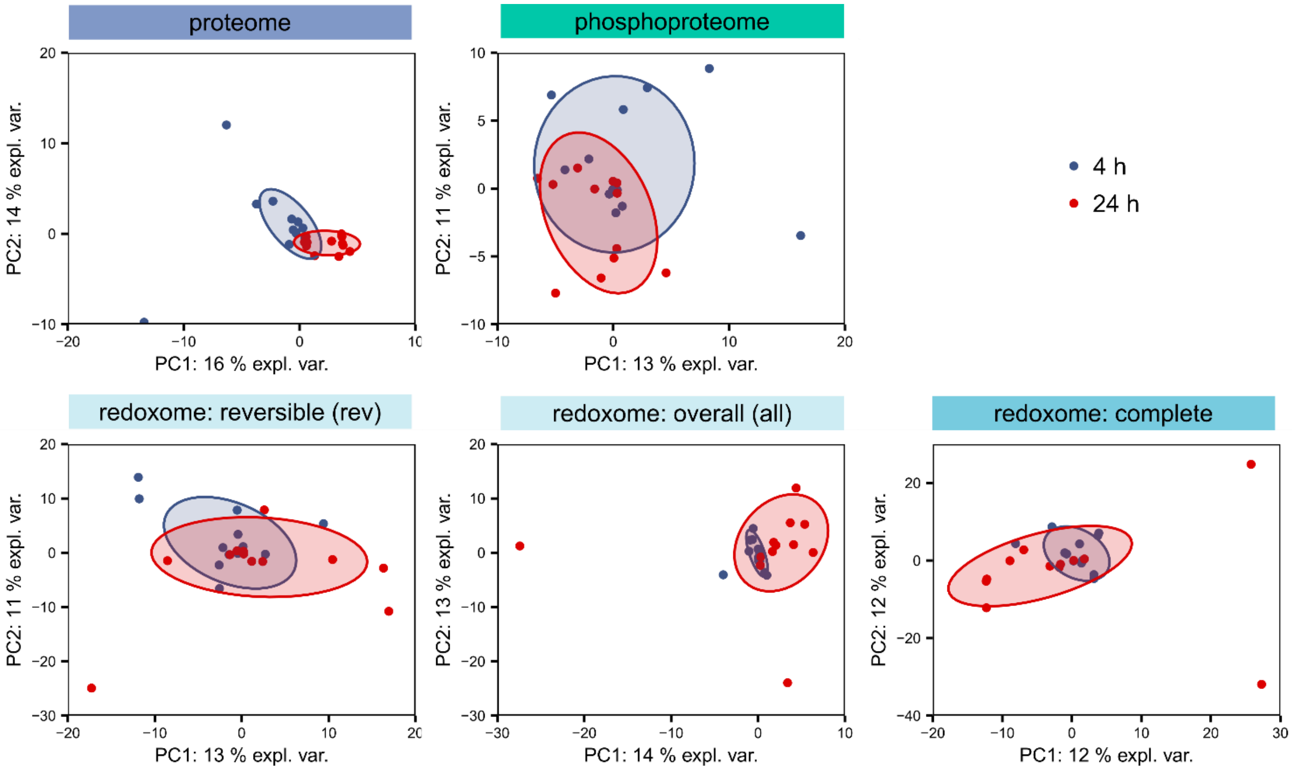

Figure S2: Principal component analyses (PCAs) of reliably identified proteins and sites. PCAs were performed with reliably quantified proteins (proteome), phosphorylation sites (phosphoproteome), reversible oxidation sites (redoxome: reversible), overall oxidation sites (redoxome: overall), and the combination of both (redoxome: complete) to assess the data reproducibility after 4 h and 24 h LPS treatment. The very low explained variances in the first two principal components suggest a good reproducibility.

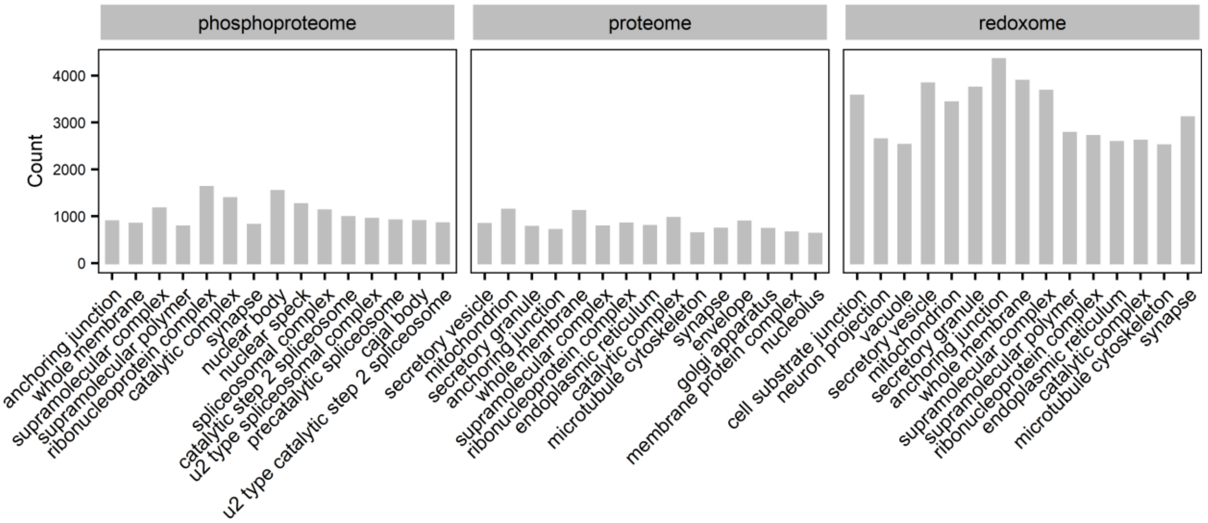

Figure S3: Assignment of identified proteins to cellular compartments.
Shown are the compartments assigned to most phosphorylation sites, proteins, or oxidation sites quantified here.

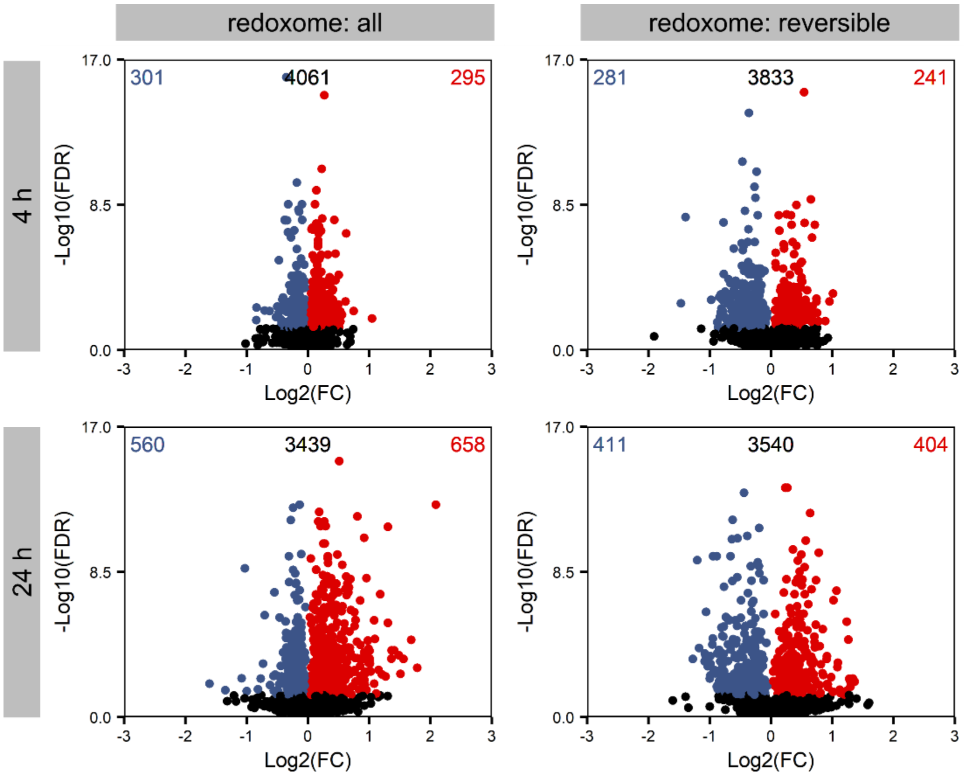

Figure S4: Affected oxidation sites.
Shown are significantly (FDR≤0.05) altered oxidation sites after LPS treatment of THP-1 macrophages for 4 h and 24 h, respectively. Overall oxidation (redoxome: all) and reversible oxidation (redoxome: reversible) are distinguished. Log2(FCs) and -Log10(FDRs) are depicted, highlighting the numbers of significantly increased (FDR≤0.05, Log2(FC)>0) or decreased (FDR≤0.05, Log2(FC)<0) sites in the corners.

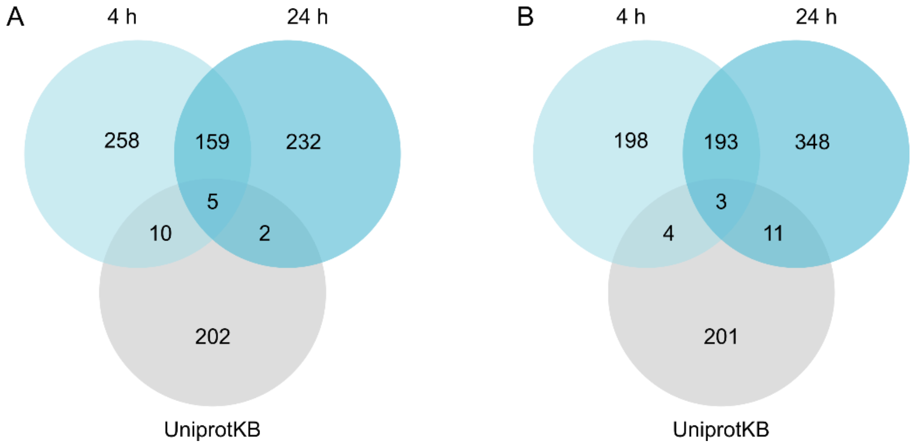

Figure S5: Overlap of affected oxidized proteins with proteins known to be modified on cysteine. Proteins showing significantly (FDR≤0.05) altered overall (A) or reversible (B) oxidation after 4 h or 24 h, respectively, were compared to proteins known for cysteine modification in general or the presence of S-nitrosocysteine in particular. For this comparison, information available in the UniProtKB was used.

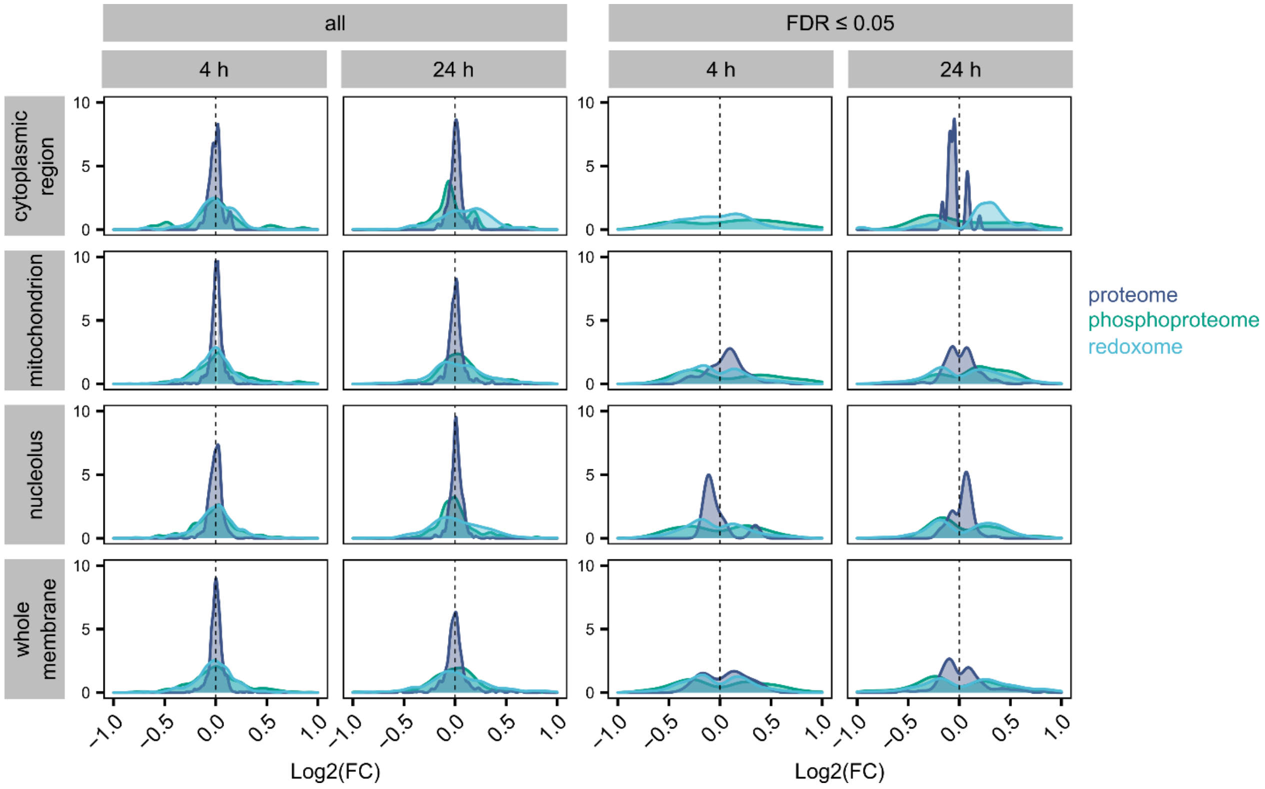

Figure S6: Compartment densities.
Shown are compartment-specific Log2(FC) distributions for all identified proteins/sites and significantly (FDR≤0.05) altered proteins/sites.

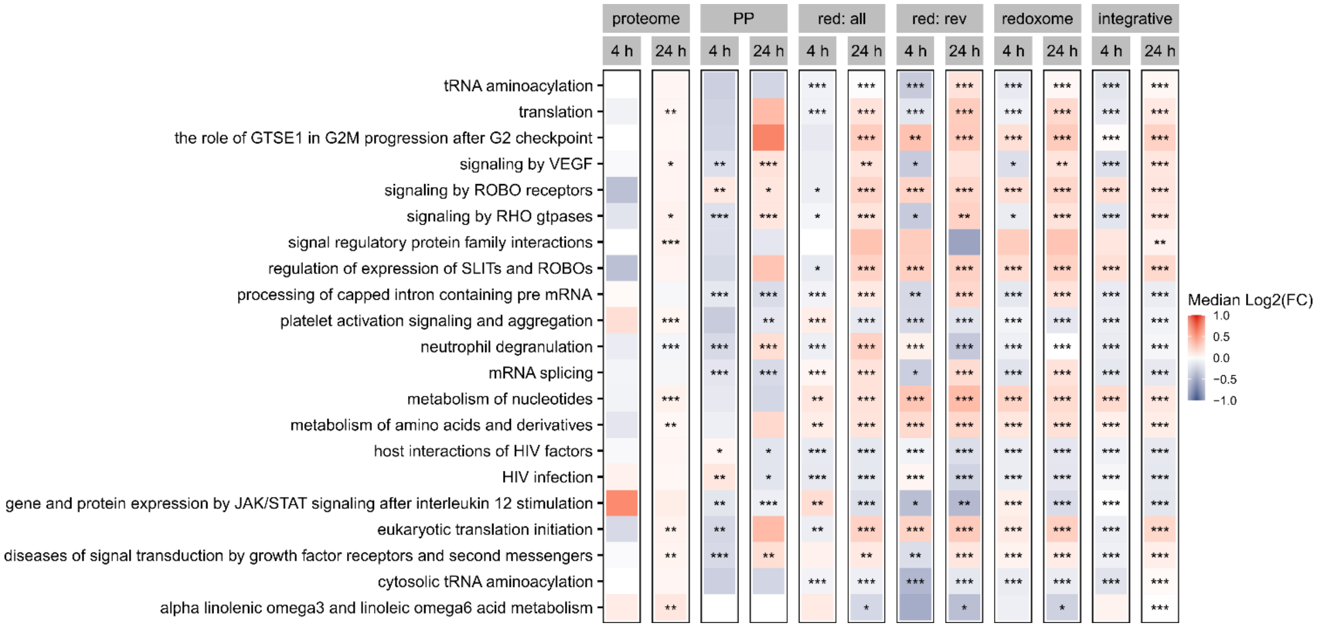

Figure S7: Top 5 significantly enriched Reactome pathways.
Combination of the top 5 Reactome pathways, significantly enriched (FDR≤0.05) either in one of the investigated omics layers or based on the integrative pathway enrichment. The significance of enrichment is provided with asterisks: * - FDR≤0.05, ** - FDR≤0.01, *** - FDR≤0.001. The color reflects the median Log2(FC) of the proteins/sites assigned to the pathway. The enrichment analysis was performed based on significantly (FDR≤0.05) altered proteins (proteome), phosphorylation sites (PP), oxidation sites in general (red: all), reversibly oxidized sites (red: rev), the combination of both types of oxidation sites (redoxome), or the combination of all three omics layers (integrative).

2 Supplementary Methods

2.1 Proteome

20 µg protein per sample was prepared for untargeted proteomics using a paramagnetic bead approach (1-3) in combination with enzymatic cleavage using trypsin and tandem mass tag (TMT, Thermo Scientific, USA) labeling as described before (4). After preparation, samples were analyzed using liquid chromatography (LC), i.e. nano-UPLC system (Ultimate 3000, Dionex, USA), coupled to a mass spectrometer (MS, QExactive HF, Thermo Scientific, USA) via a chip-based ESI source (Nanomate, Advion, USA).

2.1.1 Sample preparation on paramagnetic beads

2.1.1.1 Protein clean-up and enzymatic cleavage

20 µg per sample was used, and the volume was adjusted to 50 µl with 100 mM TEAB (Tetraethylammonium tetrahydroborate, Sigma-Aldrich, USA) first. Afterwards, samples were reduced with 5 µl 200 mM TCEP (Tris(2-carboxyethyl)phosphine hydrochloride, Sigma-Aldrich, USA) in 100 mM TEAB for 1 h at 55 °C. Subsequently, 5 µl 375 mM iodoacetamide (Merck KGaA, Germany) in 100 mM TEAB was added and incubated for 30 min at room temperature in the dark. Next, 70 µl pure acetonitrile (ACN) was added to the samples to reach a final organic content higher than 50 % (v/v), facilitating protein binding to SP3 paramagnetic beads. 2 µl SP3 carboxylated beads (SpeedBeads™ magnetic carboxylate modified particles, Cytiva, Merck KGaA, Germany) per sample were washed with water three times. After the third washing step, water was removed, and samples were added to the beads. After 8 min of protein binding to the beads, the supernatant was discarded. Then, the beads were washed twice with 200 µl 70 % (v/v) ethanol and once with 200 µl pure ACN. Finally, the proteins were digested with trypsin (1:50) for 16 h at 37 °C. Since trypsin is added in aqueous solution to the samples, the proteins are not bound to the beads during enzymatic cleavage.

2.1.1.2 TMT labeling

For TMT labeling, approximately 60 µg TMT labeling reagent (TMT 6-plex, Thermo Fisher Scientific, USA) in 5 µl pure ACN was added to the obtained peptide samples, followed by 1 h incubation at room temperature. The reaction was quenched for 15 min at room temperature by adding 1 µL 5 % (v/v) hydroxylamine in 100 mM TEAB.

2.1.1.3 Peptide clean-up

To facilitate the subsequent peptide clean-up, 140 µl pure ACN was added to each sample, resulting in an organic content higher than 95 % (v/v), enabling peptide binding to the beads. During this step, samples bearing the different TMT labels were combined. The supernatant was removed after 8 min of incubation, allowing for peptide binding to the beads. Afterwards, the samples were washed with 200 µl pure ACN. Finally, the peptides were eluted in two steps. First, with 200 µL 87 % (v/v) ACN containing 10 mM ammonium formate (pH 10), and next with two times adding 50 µL water containing 2 % (v/v) DMSO and combination of the two aqueous supernatants. Thus, two fractions of peptides were generated, evaporated and re-dissolved in water containing 0.1 % (v/v) formic acid.

2.1.2 LC-MS data acquisition

Table S1: LC gradient proteome.
Ultimate 3000, Dionex, USA
Flow rate: 0.3 µl/min, A: 0.1 % (v/v) formic acid, B: 80 % (v/v) ACN, 0.08 % (v/v) formic acid.

Time [min]	%B
**0**	4
**5**	4
**100**	30
**140**	55
**155**	99
**160**	99
**165**	4
**180**	4

Table S2: MS parameters proteome.
QExactive HF, Thermo Scientific, USA
Data-dependent acquisition

Parameter	Setting
**Polarity**	positive
**Default charge state**	2
**MS1 resolution**	120K
**MS1 AGC target**	3e6
**MS1 max IT**	120 ms
**MS1 scan range**	350 – 1550 *m/z*
**MS1 data type**	profile
**MS2 resolution**	60K
**MS2 AGC target**	1e5
**MS2 max IT**	120 ms
**TopN**	15
**Isolation window**	0.7 *m/z*
**MS2 fixed first mass**	120 *m/z*
**(N)CE**	34
**MS2 data type**	profile
**Min AGC target**	2e3
**Charge exclusion**	Unassigned, 1, >6
**Dynamic exclusion**	45 s

2.1.3 Data processing using Proteome Discoverer 2.5

Table S3: Processing workflow parameters proteome.

Parameter	Setting
**Mass analyzer**	FTMS
**MS order**	2
**Activation type**	HCD
**Precursor selection**	Use MS1 precursor
**Min precursor mass**	350 Da
**Max precursor mass**	5000 Da
**S/N threshold**	1.5
**Enzyme name**	Trypsin (Full)
**Max missed cleavages**	2
**Min peptide length**	6
**Max peptide length**	150
**Precursor mass tolerance**	10 ppm
**Fragment mass tolerance**	0.6 Da
**Dynamic modifications**	Oxidation (M), TMT6plex (peptide N-terminus), Acetyl (protein N-terminus)
**Statis modifications**	TMT6plex (K), Carbamidomethyl (C)
**FDR (strict)**	0.01
**FDR (relaxed)**	0.05

Table S4: Consensus workflow general parameters proteome.

Parameter	Setting
**Peptide confidence at least**	High
**Minimum number of peptide sequences**	2
**FDR (strict)**	0.01
**FDR (relaxed)**	0.05
**Apply strict parsimony principle**	True

Table S5: Consensus workflow quantification parameters proteome.

Parameter	Setting
**Peptides to use**	Unique + Razor
**Apply quan value corrections**	True
**Co-isolation threshold**	50
**Average reporter S/N threshold**	10
**Normalization mode**	None
**Scaling mode**	None
**For Protein Roll-Up**	Use all peptides
**Maximum allowed fold change**	100

2.2 Redoxome

20 µg protein per sample was prepared for untargeted proteomics using sequential iodoTMT labeling and the described peptide clean-up on paramagnetic beads (paragraph 2.1.1.3). After preparation, samples were analyzed using liquid chromatography (LC), i.e. nano-UPLC system (Ultimate 3000, Dionex, USA), coupled to a mass spectrometer (MS, QExactive HF, Thermo Scientific, USA) via a chip-based ESI source (Nanomate, Advion, USA).

2.2.1 Sample preparation

For the redoxome, the mixes of iodoTMT-labeled peptides were subjected to peptide clean-up on paramagnetic beads (paragraph 2.1.1.3). Only the volume had to be scaled up as described in the manuscript.

2.2.2 LC-MS data acquisition

To measure the redoxome samples, the same LC and MS parameters described for the proteome have been used (paragraph 2.1.2).

2.2.3 Data processing using Proteome Discoverer 2.5

Table S6: Processing workflow parameters redoxome.
Compared to processing proteomics samples, the IMP-ptmRS node has to be added to the workflow.

Parameter	Setting
**Mass analyzer**	FTMS
**MS order**	2
**Activation type**	HCD
**Precursor selection**	Use MS1 precursor
**Min precursor mass**	350 Da
**Max precursor mass**	5000 Da
**S/N threshold**	1.5
**Enzyme name**	Trypsin (Full)
**Max missed cleavages**	2
**Min peptide length**	6
**Max peptide length**	150
**Precursor mass tolerance**	10 ppm
**Fragment mass tolerance**	0.6 Da
**Dynamic modifications**	Oxidation (M), Acetyl (protein N-terminus)
**Statis modifications**	iodoTMT6plex (C)
**FDR (strict)**	0.01
**FDR (relaxed)**	0.05

Table S7: Consensus workflow general parameters redoxome.
Compared to processing proteomics samples, the Peptide Isoform Grouper node has to be added to the workflow. The minimum number of peptide sequences per protein has to be decreased to 1 to keep all identified peptides. Notably, analysis is performed on the peptide level, not the protein level.

Parameter	Setting
**Peptide confidence at least**	High
**Minimum number of peptide sequences**	1
**FDR (strict)**	0.01
**FDR (relaxed)**	0.05
**Apply strict parsimony principle**	True

Table S8: Consensus workflow quantification parameters redoxome.

Parameter	Setting
**Peptides to use**	Unique + Razor
**Apply quan value corrections**	True
**Co-isolation threshold**	50
**Average reporter S/N threshold**	10
**Normalization mode**	None
**Scaling mode**	None
**For Protein Roll-Up**	Use all peptides
**Maximum allowed fold change**	100

2.3 Phosphoproteome

70-100 µg protein per sample was prepared for untargeted proteomics using TMT labeling on paramagnetic beads (paragraph 2.1.1). Phosphorylated peptides were enriched using a two-step enrichment workflow. After preparation, samples were analyzed using liquid chromatography (LC), i.e. nano-UPLC system (Ultimate 3000, Dionex, USA), coupled to a mass spectrometer (MS, QExactive HF, Thermo Scientific, USA) via a chip-based ESI source (Nanomate, Advion, USA).

2.3.1 Sample preparation

For the phosphoproteome, the same paramagnetic bead approach described for the proteome (paragraph 2.1.1) has been applied. Only the elution after the peptide clean-up was done differently than for proteome and redoxome, not in two fractions but only with water. Afterwards, a two-step enrichment using a workflow based on the HighSelect™ TiO2 Phosphopeptide Enrichment Kit (Thermo Scientific, USA) and the High-Select™ Fe-NTA Phosphopeptide Enrichment Kit (Thermo Scientific, USA) was performed as described before (5).

2.3.2 LC-MS data acquisition

Table S9: LC gradient phosphoproteome.
Ultimate 3000, Dionex, USA
Flow rate: 0.3 µl/min, A: 0.1 % (v/v) formic acid, B: 80 % (v/v) ACN, 0.08 % (v/v) formic acid.

Time [min]	%B
**0**	4
**5**	4
**82.5**	18
**120**	30
**150**	55
**155**	99
**165**	99
**170**	4
**180**	4

Table S10: MS parameters phosphoproteome.
QExactive HF, Thermo Scientific, USA
Data-dependent acquisition

Parameter	Setting
**Polarity**	positive
**Default charge state**	2
**MS1 resolution**	120K
**MS1 AGC target**	3e6
**MS1 max IT**	150 ms
**MS1 scan range**	350 – 1550 *m/z*
**MS1 data type**	profile
**MS2 resolution**	60K
**MS2 AGC target**	2e5
**MS2 max IT**	150 ms
**TopN**	15
**Isolation window**	0.7 *m/z*
**MS2 fixed first mass**	120 *m/z*
**(N)CE**	34
**MS2 data type**	profile
**Min AGC target**	2e3
**Charge exclusion**	Unassigned, 1, >6
**Dynamic exclusion**	45 s

2.3.3 Data processing using Proteome Discoverer 2.5

Table S11: Processing workflow parameters phosphoproteome.
Compared to processing proteomics samples, the IMP-ptmRS node has to be added to the workflow.

Parameter	Setting
**Mass analyzer**	FTMS
**MS order**	2
**Activation type**	HCD
**Precursor selection**	Use MS1 precursor
**Min precursor mass**	350 Da
**Max precursor mass**	5000 Da
**S/N threshold**	1.5
**Enzyme name**	Trypsin (Full)
**Max missed cleavages**	2
**Min peptide length**	6
**Max peptide length**	150
**Precursor mass tolerance**	10 ppm
**Fragment mass tolerance**	0.6 Da
**Dynamic modifications**	Oxidation (M), TMTpro (peptide N-terminus), Acetyl (protein N-terminus), Phospho (S, T, Y)
**Statis modifications**	TMTpro (K), Carbamidomethyl (C)
**FDR (strict)**	0.01
**FDR (relaxed)**	0.05

Table S12: Consensus workflow general parameters phosphoproteome.
Compared to processing proteomics samples, the Peptide Isoform Grouper node has to be added to the workflow. The minimum number of peptide sequences per protein has to be decreased to 1 to keep all identified peptides. Notably, analysis is performed on the peptide level and not on the protein level .

Parameter	Setting
**Peptide confidence at least**	High
**Minimum number of peptide sequences**	1
**FDR (strict)**	0.01
**FDR (relaxed)**	0.05
**Apply strict parsimony principle**	True

Table S13: Consensus workflow quantification parameters phosphoproteome.

Parameter	Setting
**Peptides to use**	Unique + Razor
**Apply quan value corrections**	True
**Co-isolation threshold**	50
**Average reporter S/N threshold**	10
**Normalization mode**	None
**Scaling mode**	None
**For Protein Roll-Up**	Use all peptides
**Maximum allowed fold change**	100

3 References

1. Hughes CS, Foehr S, Garfield DA, Furlong EE, Steinmetz LM, Krijgsveld J. Ultrasensitive proteome analysis using paramagnetic bead technology. Mol Syst Biol. 2014;10:757.

2. Hughes CS, Moggridge S, Muller T, Sorensen PH, Morin GB, Krijgsveld J. Single-pot, solid-phase-enhanced sample preparation for proteomics experiments. Nat Protoc. 2019;14:68–85.

3. Bannuscher A, Karkossa I, Buhs S, Nollau P, Kettler K, Balas M, et al. A multi-omics approach reveals mechanisms of nanomaterial toxicity and structure–activity relationships in alveolar macrophages. Nanotoxicology. 2019:1-15.

4. Wang Z, Karkossa I, Großkopf H, Rolle-Kampczyk U, Hackermüller J, von Bergen M, et al. Comparison of quantitation methods in proteomics to define relevant toxicological information on AhR activation of HepG2 cells by BaP. Toxicology. 2021;448:152652.

5. Großkopf H, Walter K, Karkossa I, von Bergen M, Schubert K. Non-Genomic AhR-Signaling Modulates the Immune Response in Endotoxin-Activated Macrophages After Activation by the Environmental Stressor BaP. Frontiers in Immunology. 2021;12(1008).
